# What do users in a polycystic ovary syndrome (PCOS) forum think about the treatments they tried: Analysing treatment sentiment using machine learning

**DOI:** 10.1007/s13246-025-01539-9

**Published:** 2025-04-14

**Authors:** Rebecca H. K. Emanuel, Paul D. Docherty, Helen Lunt, Rebecca E. Campbell

**Affiliations:** 1 Department of Mechanical Engineering, University of Canterbury, Christchurch, New Zealand; 2 Institute for Technical Medicine, Furtwangen University, Villingen-Schwenningen, Germany; 3 Diabetes Services, Health New Zealand, Canterbury, New Zealand; 4 School of Biomedical Sciences, Department of Physiology, Centre for Neuroendocrinology, University of Otago, Dunedin, New Zealand

**Keywords:** PCOS, Machine learning, Internet research, Endocrinology

## Abstract

Polycystic ovary syndrome (PCOS) is a heterogenous condition that is estimated to effect up to 21% of reproductive aged people with ovaries. In previous work, a dataset of PCOS features was derived from approximately 100,000 PCOS subreddit users via machine learning. In this study, an exploration of treatment response within the PCOS subreddit was undertaken with the derived dataset. The treatment or symptom features in the dataset had sentiment labels indicating when a treatment was perceived to improve or worsen a condition or symptom. When different features were mentioned within two sentences of each other without conflicting sentiment, it could be assumed that they were related. This assumption allowed for a broad analysis of the perceived effect of popular treatments on the most frequently mentioned symptoms. In general, lifestyle changes and supplements were the most positively regarded, while contraceptives were frequently associated with considerable negative sentiment. For PCOS weight loss, unspecified dieting (RR 5.19, 95% CI 3.28–8.19, n = 99) and intermittent fasting (RR 33.50, 95% CI 8.54–131.34, n = 69) were the most successful interventions. Inositol was associated with a large range of favourable outcomes and was one of the few treatments associated with improved mental health [depression (RR 4.25, 95% CI 1.72–10.51, n = 21), anxiety (RR 5.83, 95% CI 2.76–12.35, n = 41) and mood issues (RR 25.00, 95% CI 3.65–171.10, n = 26)]. Combined oral contraceptive pills as a whole were strongly associated with adverse effects such as worsening depression (RR 0.06, 95% CI 0.02–0.25, n = 33), anxiety (RR 0.10, 95% CI 0.03–0.36, n = 23), fatigue (RR 0, n = 45) and low libido (RR 0.03, 95% CI 0.01–0.24, n = 30). However, combined contraceptives with anti-androgenic progestins were associated with more favourable experiences. This study demonstrates the utility of machine learning to derive measurable patient experience data from an internet forum. While patient experience data derived using machine learning is not a substitute for traditional clinical trials, it is useful for mass validation and hypothesis generation. This paper may serve as the first exploration into this category of clinical internet forum research.

## Introduction

Polycystic ovary syndrome (PCOS) is an endocrine condition prevalent in people with ovaries that can affect both the metabolic and reproductive systems [1, 2]. Current international guidelines support the use of the Rotterdam criteria for PCOS diagnosis, which requires two of three features [3]. The first feature is anovulation or oligo-ovulation [4]. Signs of ovulation irregularities can include menstrual irregularities, infrequent menstruation, a lack of menstruation or overly frequent menstruation [3]. The second feature is biochemical or clinical hyperandrogenism [4]. Biochemical hyperandrogenism can be indicated by high levels of serum androgens, such as testosterone or dehydroepiandrosterone-sulphate (DHEA-S) [3]. Clinical hyperandrogenism is typically indicated by hirsutism [3]. Alopecia and excessive acne can also be signs of clinical hyperandrogenism [3]. The third criteria is evidence of polycystic ovarian morphology [4]. PCOS is also closely associated with a number of other conditions, including insulin resistance [5], obesity [6], pregnancy complications [7, 8], infertility [9], and poor mental health [10].

Patient surveys reveal widespread dissatisfaction with PCOS treatment [11, 12], while clinicians have expressed concern regarding the lack of supporting evidence for various treatments [11]. Current PCOS treatment options include prescribed insulin sensitisers, such as metformin, and anti-androgens, such as spironolactone [3]. Combined oral contraceptives are also considered in the treatment of menstrual irregularities and hyperandrogenism [3]. Lifestyle changes such as dieting or exercise plans have been shown to improve PCOS symptoms [13, 14], and are often considered first line treatments. Finally, dietary supplements are emerging as possible interventions [15]. Researchers are increasingly recommending individualised treatment plans for people with PCOS [16]. However, there is a paucity of research guiding how different clinical symptoms respond to different interventions.

In this study, data from a PCOS internet forum, extracted and labelled using machine learning, was used to determine treatment outcome perceptions. Previous work has explored the laboratory test results posted within the PCOS internet forum [17, 18]. A summary of how the different PCOS features were identified within the internet forum was detailed in previous work [19]. The current study explores how different treatments identified within the forum were associated with the improvement or worsening of different clinical symptoms.

## Methods

Approximately 85,000 posts and 630,000 comments, posted before March 2023, were downloaded from the PCOS subreddit [20] using the Pushshift Reddit dataset [21]. Utilising this data for this research was considered out-of-scope for review by the University of Canterbury ethics committee. This was predominantly due to the public availability, voluntary posting and anonymised nature of the posts [17].

Despite the data being publicly available, many consider it the researcher’s responsibility to maximise their own ethical approach to its use [22–24]. Identification is probably the most serious risk to internet users involved in this kind of public data research [25]. For a survey of 21 people, 70% of people who posted online to ask for medical advice were primarily concerned with anonymity [26]. This study aimed to preserve anonymity of the reddit users by not including names or quotes from individual reddit posters, and using aggregated data in any publication. The number of discrete posts was sufficiently large that identification of individuals in relation to their unique content is extremely unlikely. Furthermore, no content from individual users was singled out or highlighted in this research.

Unfortunately, the scale of this study and nature of the research made it unfeasible to gain informed consent from the reddit users. The Association of Internet Researchers (AoIR) recommends seeking informed consent from those quoted in research, and no such quotation exists in this research. Effort was made to only include participants who consented to their information being shared on the PCOS subreddit by removing data describing someone else’s PCOS-related experience.

The posts and comments were created by approximately 100,000 unique users. Using the limited manual labelling and machine learning methods detailed in previous work [19], a database of users and their declared features was created. Features could be symptoms, treatments, diagnosis status, or other pieces of information relevant to PCOS. The most frequent features were also labelled with perceived improving, neutral or worsening sentiment. In this context, sentiment labelling refers to identifying positive, negative or neutral sentiments within text. Treatment features were labelled as ‘improving’ when a user stated they thought the treatment was helping with their condition. They were labelled as ‘worsening’ when a user stated the treatment was either making their condition worse or failing to help their condition as intended. Neutral sentiments were also labelled when a treatment was noted, but sentiment was absent or ambiguous. Symptom features were labelled with sentiment according to Table 1.

**Table 1:** The different condition labels relevant in this study and an explanation of their corresponding sentiments

Condition Label	Description/Neutral	Improving	Worsening
Body Weight	Mention of struggling with unwanted increased body weight	Mention of deliberate weight loss or successful weight maintenance	Mention of unwanted weight gain or failure to lose weight
Menstrual Irregularities	Mention of experiencing menstrual irregularities	Mention of menstrual irregularities improving	Mention of menstrual irregularities worsening or failing to improve
Oligo-Ovulation	Mention of experiencing eight or fewer periods a year or menstrual cycles longer than 35 days	Mention of menstrual cycles getting more frequent	Mention of menstrual cycles getting less frequent or failing to get more frequent
Amenorrhea	Mention of a lack of menstrual cycle for at least three months	Mention of menstrual cycles returning	Mention of menstrual cycles failing to return
Menorrhagia	Mention of menstrual bleeding that lasts more than seven days	Mention of menstrual bleeding ceasing	Mention of menstrual bleeding failing to cease
Polymenorrhea	Mention of menstrual cycles shorter than 21 days	Mention of menstrual cycles getting less frequent	Mention of menstrual cycles getting more frequent or failing to get less frequent
Hirsutism	Mention of too much body or facial hair	Mention of decreasing unwanted hair growth rate or coverage	Mention of increasing unwanted hair growth rate or coverage or failure to decrease unwanted hair
Alopecia	Mention of thinning or balding head hair	Mention of decreased head hair thinning or balding	Mention of increased head hair thinning or balding or failure to decrease thinning or balding
Acne	Mention of too much acne on the face or body	Mention of decreased acne frequency or severity	Mention of increased acne frequency or severity or failure to decrease acne
Depression	Mention of struggling with depression	Mention of decreased depressive symptoms	Mention of increased depressive symptoms or failure to decrease symptoms
Anxiety	Mention of struggling with anxiety or experiencing panic attacks	Mention of decreased anxiety symptoms	Mention of increased anxiety symptoms or failure to decrease symptoms
Mood Issues	Mention of experiencing mood swings or excessive irritability	Mention of decreased mood issues	Mention of increased mood issues or failure to decrease issues
Head Pain	Mention of frequent or persistent headaches or migraines	Mention of decreased head pain frequency or severity	Mention of increased head pain frequency or severity or failure to decrease head pain
Cravings	Mention of frequent or severe hunger or food cravings	Mention of decreased hunger frequency or severity	Mention of increased hunger frequency or severity or failure to decrease hunger
Irritated Bowel	Mention of symptoms of an irritated bowel such as bloating, cramping, diarrhoea or constipation	Mention of decreased gastrointestinal symptoms	Mention of increased gastrointestinal symptoms or failure to decrease symptoms
Bloating	Mention of frequent or severe abdominal bloating	Mention of decreased bloating frequency or severity	Mention of increased bloating frequency or severity or failure to decrease bloating
Cramping	Mention of frequent or severe abdominal cramping	Mention of decreased cramping frequency or severity	Mention of increased cramping frequency or severity or failure to decrease cramping
Inflammation	Mention of frequent or severe perceived external inflammation such as redness or swelling on their skin	Mention of decreased inflammation frequency or severity	Mention of increased inflammation frequency or severity or failure to decrease inflammation
Fatigue	Mention of frequent or severe exhaustion, tiredness, fatigue, or a consistent lack of energy	Mention of decreasing fatigue	Mention of increasing fatigue or failure to decrease fatigue
Low Libido	Mention of struggling with a low libido	Mention of libido increasing	Mention of libido decreasing or failing to increase

The performance of machine learning models was assessed using manually labelled testing datasets. Overall, the vast majority of feature classifiers performed with precision above 85% and the majority of sentiment classifiers performed with accuracy above 90% [19]. These levels of precision and accuracy are generally considered good for complex text classification machine learning and were deemed appropriate for the current application [19]. Appendix A provides performance metrics for the machine learning models relevant to this study.

When two identified treatment or symptom features were mentioned within two sentences of each other, it was assumed that the features were being discussed in relation to each other. This allowed the generation of inferences about the relationships between different treatments and symptoms. If sentiment labels attached to the two features were contradictory, then the assumption was abandoned. The assumptions made regarding different sentiment combinations for the treatment-symptom pairs are shown in Table 2. For example, if, within a few sentences, someone mentioned that they perceived spironolactone was helpful to them and that their menstrual irregularities improved, the assumption would be made that the person perceived taking spironolactone improved their menstrual irregularities. If however, the spironolactone was associated with worsening sentiment, then it was assumed the spironolactone use was not mentioned in relation to the improving menstrual irregularities. The two sentence limit was imposed due to the multi-themed, unstructured nature of the text posts and comments. This two sentence limit increased the likelihood that two features were being discussed in relation to each other.

**Table 2:**
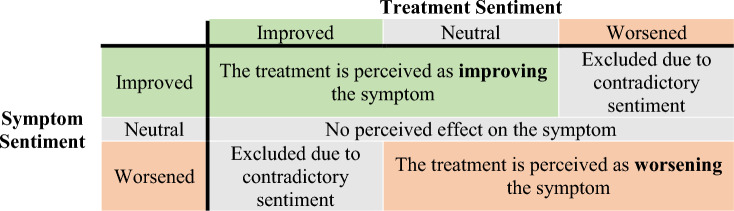
How the different treatment and symptom sentiment combinations led to different assumptions

Risk ratios ($RRs$) were calculated using the number of instances that a treatment improved a symptom (${n}_{I}$) and the number of instances that a treatment worsened a symptom (${n}_{W}$). This is shown in Eq. 1. Confidence intervals for 95% confidence were also calculated (Eq. 2).

1 $$RR = \frac{{n_{1} }}{{n_{w} }}$$

2 $$CI = \exp \left[ {In(RR) \pm 1.9599\sqrt {\frac{1}{{n_{1} }} + \frac{1}{{n_{w} }} - \frac{2}{{(n_{1} + n_{w} )}}} } \right]$$

Within the dataset, there were 75 symptom labels and 157 treatment labels that had available sentiment information. Overall, 3245 symptom-treatment combinations were discussed by users with varying sentiment. Only symptom-treatment combinations with more than 20 users discussing relevant sentiment were included in the analysis. This was to avoid presenting combinations with weak supporting data.

The CIs were used to assess the statistical significance of the result for each combination. If CIs encompassed 1.0 for the absolute RR, then the sentiment found was deemed non-significant. It is important to note that this study was exploratory rather than hypothesis driven. Therefore, no statistical adjustments were made for the multiple combinations being explored. As such, individual results from this study should not be considered in isolation.

## Results

It was possible to glean information about both the overall perceptions towards specific treatments within the PCOS subreddit, and associations between treatments and different symptoms. Throughout the results and discussion, treatments have been grouped into four categories. Prescribed treatments are treatments that cannot be obtained without a prescription from a registered health practitioner in most high and middle income countries, excluding contraceptives. Contraceptive treatments are treatments that are commonly used as contraceptives. Lifestyle changes are diets or exercise regimens. Supplements are treatments that can be purchased without a prescription. Melatonin is inconsistently regulated internationally [27], and has been grouped with the supplements for this study.

### Overall sentiments

**Fig. 1:**
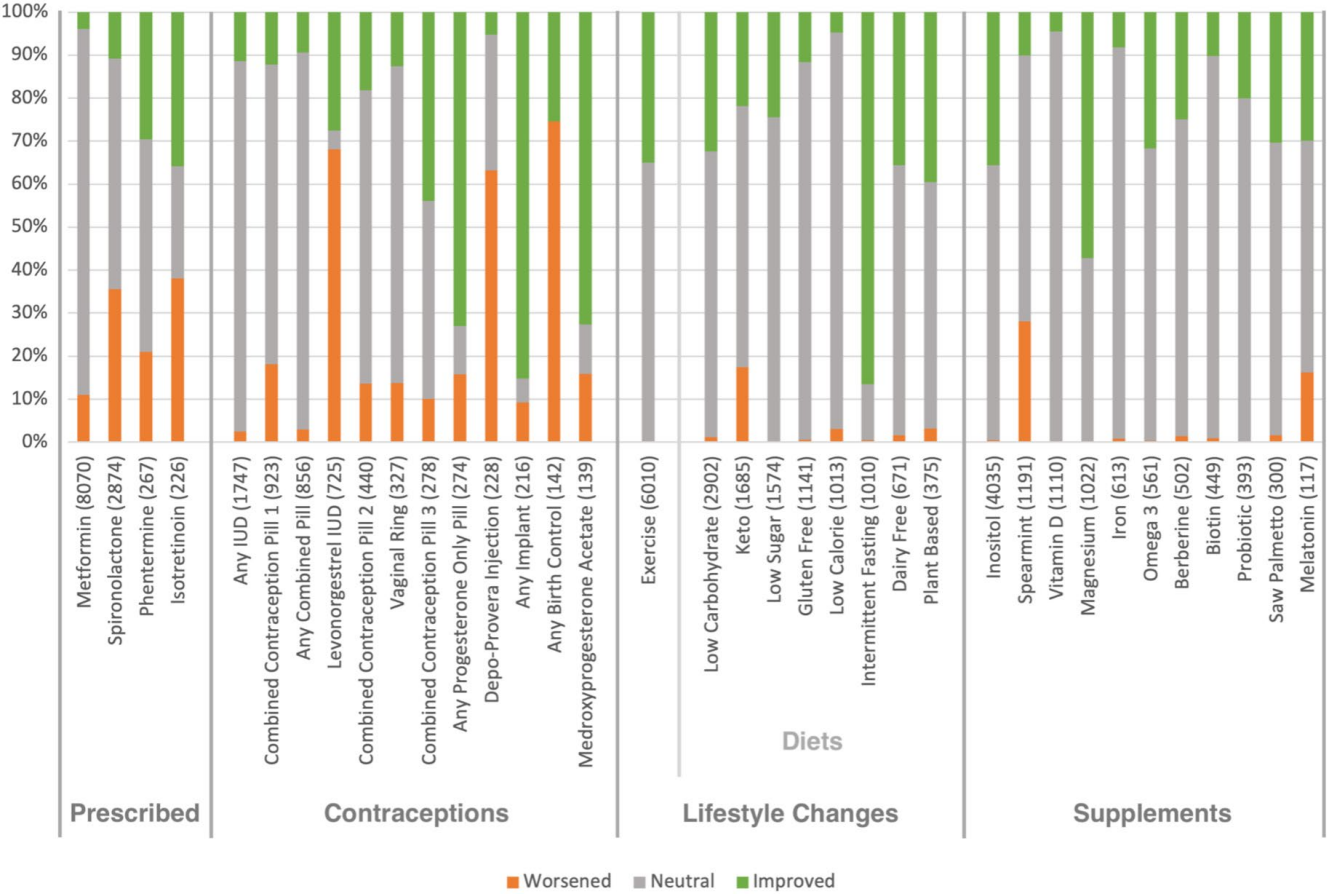
Overall sentiments for different interventions. IUD, intra uterine device; Combined Contraception Pill 1 contained 3 mg of drospirenone and 0.02 mg of ethinyl estradiol; Combined Contraception Pill 2 contained 3 mg of drospirenone and 0.03 mg of ethinyl estradiol; Combined Contraception Pill 3 contained 2 mg cyproterone acetate and 0.035 mg of ethinyl estradiol. The number of users who commented on each intervention within the dataset is shown in parentheses. Only treatments with more than 100 users commenting on them were included

**Fig. 2:**
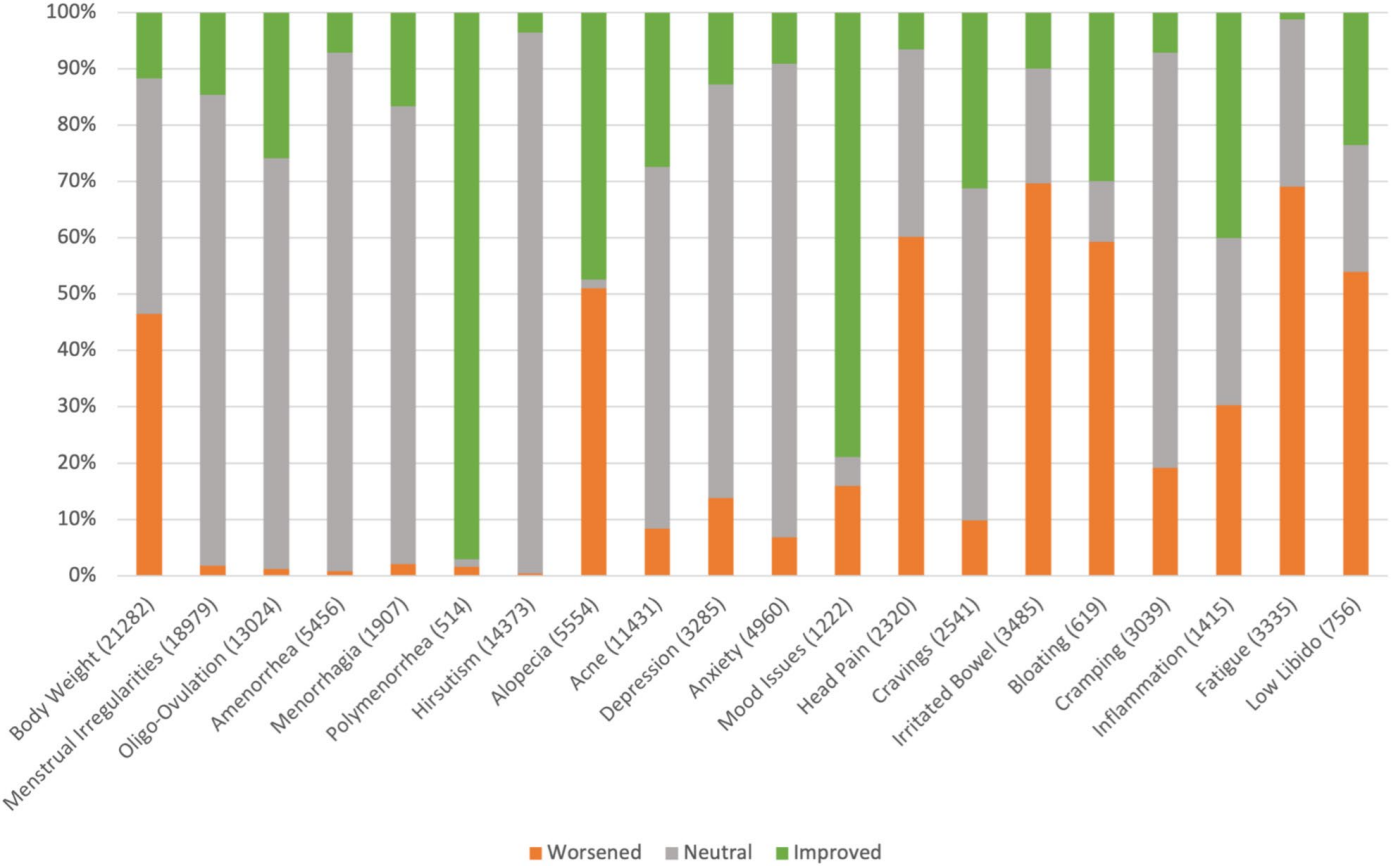
Overall sentiments for different conditions. Conditions defined in Table 1. The number of users who commented on each symptom within the dataset is shown in parentheses

Figure 1 shows the ratios of improving, worsening and neutral sentiments across the treatment labels with more than 100 user sentiments. Figure 2 shows the ratios of perceptions for common conditions.

### Treatment combinations

**Fig. 3:**
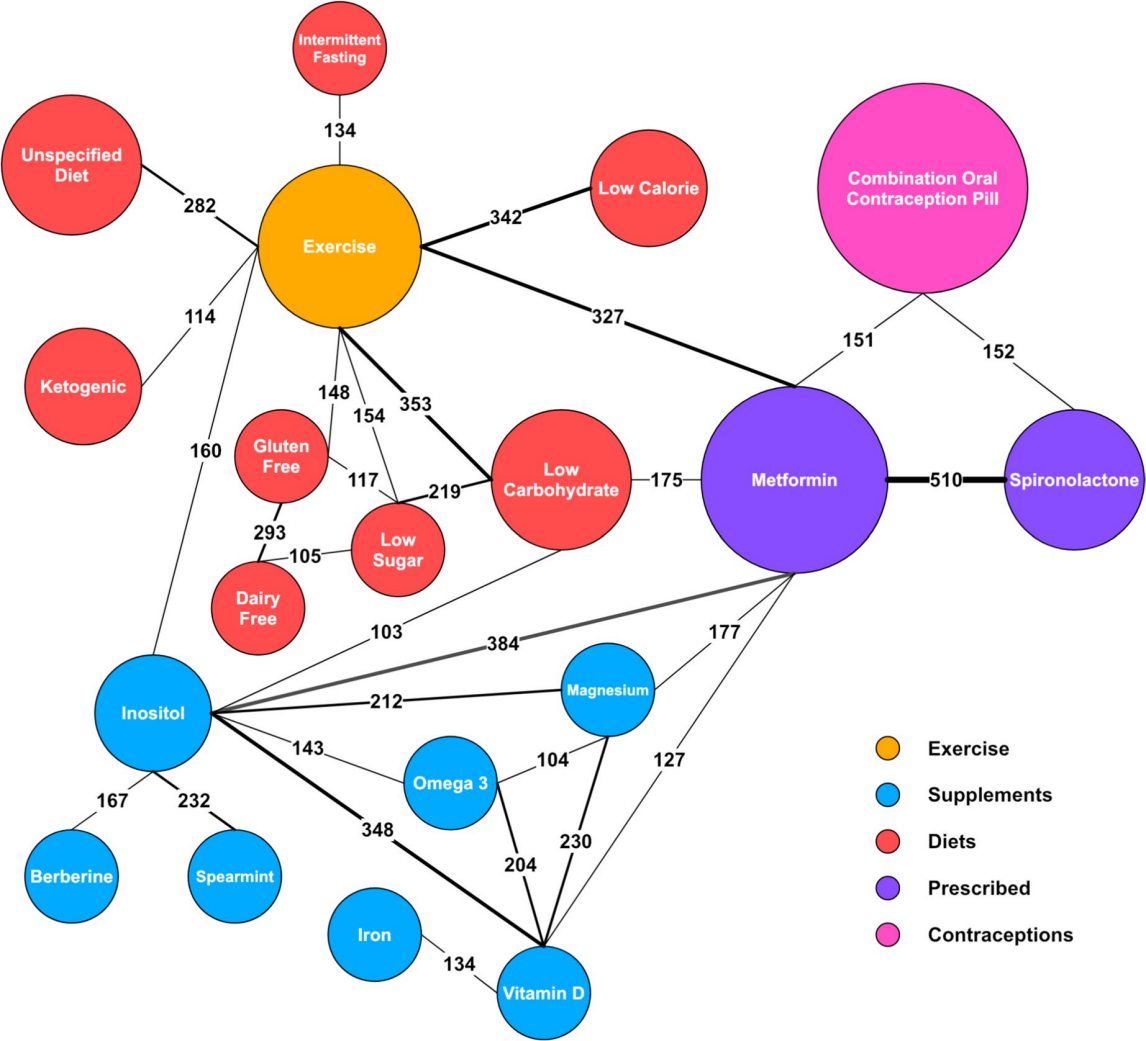
Network diagram of treatment combinations. Circle size is proportional to treatment prevalence, line thickness is proportional to number of combinations, and numerals on lines specify the number of combinations

A network diagram was created to show the common treatment combinations (Fig. 3).

### Treatment responses

This study yielded a substantial volume of treatment outcome results for 31 different treatments. Of these 31 results, 5 were prescribed treatments, 7 were hormonal contraceptives, 10 were lifestyle changes, and 9 were dietary supplements. In this study, the most significant results and trends are reported below. Other publications will explore the individual results with respect to the existing academic literature in more detail, as doing so in a single paper would be impractical.

#### Body weight, cravings and inflammation

Intermittent fasting (RR 33.50, 95% CI 8.54–131.34, n = 69) and unspecified dieting (RR 5.19, 95% CI 3.28–8.19, n = 99) had the most positive effects on body weight within the PCOS subreddit, and were in fact the only treatments perceived to assist with weight loss. Levonorgestrel IUDs (intra uterine devices) (RR 0.01, 95% CI 0.002–0.10, n = 72), medroxyprogesterone acetate injections (RR 0.05, 95% CI 0.01–0.18, n = 45), and combined oral contraceptive pills (COCPs) in general (RR 0.03, 95% CI 0.01–0.05, n = 305) had the poorest body weight outcomes. Exercising (RR 13.00, 95% CI 3.41–49.62, n = 28) and inositol supplementation (RR 10.00, 95% CI 4.65–21.53, n = 66) seemed most effective for reducing excessive hunger, and exercising was by far the most associated treatment label with reduced inflammation (RR 20.50, 95% CI 5.29–79.46, n = 43).

#### Menstrual irregularities

A large proportion of treatments were associated with improving menstrual irregularities. Lifestyle changes and supplements had the most positive sentiments [low carbohydrate diet (RR inf, n = 107), exercise (RR inf, n = 82), spearmint tea (RR inf, n = 25), and inositol supplementation (RR 69.50, 95% CI 26.27–183.90, n = 282)], while hormonal contraceptives and prescribed treatments had fewer, but were still reported to improve menstrual irregularities overall [progesterone only pills (RR 3.2, 95% CI 1.44–7.13, n = 21), COCPs (RR 3.33, 95% CI 1.60–6.94, n = 26), metformin (RR 15.08, 95% CI 8.90–25.55, n = 209) and spironolactone (RR 3.38, 95% CI 1.79–6.37, n = 35)]. Improvement towards oligo-ovulation specifically was much the same as menstrual irregularities overall. Inositol and metformin were associated with improving polymenorrhea [inositol (RR inf, n = 48) and metformin (RR inf, n = 27)] and amenorrhea [inositol (RR 38.00, 95% CI 5.49–263.19, n = 39) and metformin (RR 21.00, 95% CI 3.09–142.81, n = 22)] specifically. Inositol was the only intervention associated with improving menorrhagia (RR 21.00, 95% CI 3.09–142.81, n = 22).

#### Hyperandrogenism

Spironolactone (RR inf, n = 30) and inositol (RR inf, n = 21) seemed most effective for the treating hirsutism. Dieting (RR inf, n = 24), minoxidil (RR 11.50, 95% CI 3.03–43.67, n = 25) and saw palmetto (RR 5.00, 95% CI 2.21–11.31, n = 30) seemed most effective for improving alopecia, while COCPs appeared to worsen alopecia (RR 0.22, 95% CI 0.14–0.34, n = 94). Many treatments reportedly improved acne, with cyproterone acetate containing COCPs (RR inf, n = 24) and a dairy free diet (RR 54.00, 95% CI 7.74–376.65, n = 55) appearing to be the most effective.

#### Mental health

Inositol supplementation seemed to have the most positive effect on mental health in the PCOS subreddit with improved depression (RR 4.25, 95% CI 1.72–10.51, n = 21), anxiety (RR 5.83, 95% CI 2.76–12.35, n = 41) and mood issues (RR 25.00, 95% CI 3.65–171.10, n = 26). COCPs were most associated with decreased mental health, showing worsening depression (RR 0.06, 95% CI 0.02–0.25, n = 33) and anxiety (RR 0.10, 95% CI 0.03–0.36, n = 23).

#### Adverse effects

The treatments associated with the most significant adverse effects were metformin [head pain (RR 0.02, 95% CI 0.01–0.08, n = 98), bowel irritation (RR 0.03, 95% CI 0.01–0.04, n = 523), bloating (RR 0.47, 95% CI 0.25–0.88, n = 25), cramping (RR 0.20, 95% CI 0.08–0.50, n = 24) and fatigue (RR 0.02, 95% CI 0.004–0.06, n = 135)], spironolactone [head pain (RR 0.04, 95% CI 0.01–0.25, n = 29), bowel irregularities (RR 0.14, 95% CI 0.06–0.36, n = 32) and fatigue (RR 0.04, 95% CI 0.01–0.26, n = 27)], and COCPs [head pain (RR 0.01, 95% CI 0.002–0.09, n = 76), bowel irritation (RR 0.03, 95% CI 0.004–0.18, n = 39), fatigue (RR 0, n = 45) and low libido (RR 0.03, 95% CI 0.01–0.24, n = 30)]. Diets restricting carbohydrates were also significantly associated with fatigue [low carbohydrate (RR 0.05, 95% CI 0.01–0.21, n = 39) and ketogenic (RR 0, n = 22)].

## Discussion

### Overall sentiments and treatment combinations

The overall treatment sentiments in Fig. 1 show the most positively regarded treatments with negligible negative sentiment appeared to be intermittent fasting and magnesium supplementation. In general, lifestyle changes and supplements had the lowest rate of negative sentiment, potentially due to minimal adverse effects when compared to prescribed treatments and contraceptives. Contraceptives often had significant negative sentiment, especially medroxyprogesterone acetate injections and levonorgestrel IUDs. Adverse effects from prescribed treatments also led to significant negative sentiments.

The overall symptom sentiments in Fig. 2 show that certain conditions such as hirsutism had very limited sentiment detected. Others, such as fatigue, rarely had improvement detected, while menstrual irregularities rarely had worsening detected. The primary driving factor towards these biases was scarcity of examples within the subreddit training data. If very few people mentioned a symptom had improved or worsened, then these sentiments did not appear often in the machine learning training data, and did not lead to well performing machine learning detection. In particular, hirsutism is a difficult condition for the layperson to detect changes in and slow to change, thus not as many people reported whether their hirsutism was improving or getting worse in the training dataset. Similarly, few people reported their fatigue improving or their menstrual irregularities getting worse.

The network diagram displaying commonly paired treatments shows that the most common treatment combination was metformin and spironolactone (Fig. 3). The association of these drugs was expected, as they could be considered the most recommended treatments for PCOS outside of hormonal contraceptives and lifestyle changes [3], and they each target a different side of PCOS dysregulation. Other particularly common combinations included metformin and exercise, low carbohydrate diets and exercise, low calorie diets and exercise, inositol and metformin, and inositol and vitamin D. Combining diets with exercise is common and expected as either alone is not as effective at changing body composition [28]. Inositol and metformin are both insulin sensitisers and have been shown to work well in combination [29]. Inositol, exercise, vitamin D and metformin were all commonly used in combination with other treatments. For the first three interventions, this could be because they are generally beneficial and can be implemented without input from a health care professional.

### Treatment responses

Many of the key results in this study are at least partially supported by the literature. Intermittent fasting has been shown to facilitate weight loss, and may also have a beneficial effect on insulin resistance and hyperandrogenism in PCOS [30–32]. Likewise, meta-analyses exploring PCOS and dieting in general have concluded that dietary interventions decreased body weight and improved metabolic health [14, 33]. The subreddit association between dairy intake and increased acne has also been validated by a meta-analysis exploring this [34]. Additionally, the varied benefits of exercise towards improving PCOS are well documented within the literature [13, 14, 35–37].

The effect of contraceptives on body weight and metabolism is more controversial [38]. However, some studies found that COCPs are associated with poorer metabolic outcomes [39–41], and increased body weight or excessive hunger are often reported as adverse effects [40, 42]. There is also evidence to suggest that certain androgenic progestins may worsen metabolic issues [38, 43]. Our findings that certain contraceptives may worsen body weight, but contraceptives with anti-androgenic progestins may be beneficial supports these ideas. The effect of COCPs on mental health was also controversial, with meta-analyses of clinical studies finding the COCPs neither improved nor worsened mental health in the general population [44, 45]. However, when people report side effects of COCPs, they often report increased depression, anxiety or mood swings [38, 40, 42, 46]. A meta-analysis of cohort studies also found an increased risk of suicide in people on COCPs in comparison to people not taking them [47]. It is possible that certain subsets of people are particularly at risk of COCPs decreasing their mental health [48], and the PCOS subreddit data implies that people with PCOS may be one of these subsets. However, existing research related to the effect of PCOS and COCPs on mental health is limited [49].

Inositol supplementation was associated with a lot of positive outcomes within this study. An umbrella review of 28 meta-analyses found that inositol supplementation may improve insulin resistance, hyperandrogenism, and ovulation rate in PCOS [50]. The perception in the subreddit that inositol supplementation can improve the mental health of people with PCOS concurs with a literature review exploring that specific relationship [51]. However, there has recently been concern surrounding the integrity of some key studies underpinning inositol research within the literature [52]. As such, the existing inositol research could be considered too limited to draw conclusions [52].

Spironolactone is a common anti-androgen for the treatment of PCOS [3] and has been shown to improve hyperandrogenism [40, 53–55] and menstrual irregularities [40, 54] in PCOS. Thus, it makes sense that the subreddit considered it one of the best treatments for hyperandrogenism. Similarly, metformin is often recommended to improve PCOS [3], and has been shown to improve PCOS insulin resistance [40, 56–58], menstrual irregularities [40, 57, 59–61] and hyperandrogenism [40, 57–59, 62, 63] in the literature. However, adverse effects such as those described in the PCOS subreddit were also present in the literature for spironolactone [64–66] and metformin [58–61, 63, 67–69].

Some of the key results in this study had much smaller bodies of literature to validate them. This was primarily due to a lack of exploration, rather than the presence of studies showing contradictory effects. The PCOS subreddit demonstrated a strong association between saw palmetto supplementation and improved alopecia. The authors’ did not find any conclusive evidence of saw palmetto’s effect on PCOS alopecia, however, there was research indicating that saw palmetto may improve hair loss in general [70, 71]. Likewise, the PCOS subreddit indicated that spearmint tea may improve menstrual irregularities, but only two studies were found that explored spearmint tea in relation to females with hyperandrogenism [72, 73]. Both found that spearmint tea decreased hyperandrogenism [72, 73], which may improve menstrual irregularities due to the interconnected nature of PCOS dysregulation [74].

### Methodology and limitations

Using machine learning to extract medical information from internet forums is a comparatively new and interesting source of clinical data. Previous work has shown that the PCOS subreddit population contains clinically relevant PCOS phenotypes [17, 18]. As previously noted, the machine learning used to identify the different PCOS features and sentiments had high levels of precision and accuracy [19]. Furthermore, the wealth of data available resulted in broad and detailed information regarding a range of treatments.

However, as with any study, this data gathering approach had several limitations. Real-world research, such as the research done in this study, is not a substitute for controlled clinical trials. It is useful for gaining detailed sentiment information across a broad spectrum of related features, but there is no way to control for user biases and the placebo/nocebo effect. Conversely, randomised clinical trials with blinding help reduce the effects of researcher biases, subject biases, and the placebo/nocebo effect. They can also better isolate specific treatments in a controlled environment. Thus, they are more able to ascertain the true effect of a treatment. However, clinical trials can be biased in their own ways by sample population availability and exclusion criteria. The data obtained in this study describes the sentiments associated with different treatments and offers a foundation to form future hypotheses, but it is not able to confirm the effectiveness of treatments in the same way that randomised controlled trials can. Rather, real-world data such as ours can strengthen the findings of traditional clinical data by complimenting them, and vice versa [75].

The data from the PCOS subreddit encompasses all people who chose to participate in the subreddit discussion on their own behalf. This includes people with diagnosed PCOS and people who suspect they may have PCOS. A recent international guideline (‘clinical research priority roadmap’) noted discrepancies between conducted PCOS research and end-user priorities [76]. Historically, PCOS clinical trials typically recruit participants with clearly defined PCOS features, or a subset of PCOS features, thus participant characteristics might not reflect the characteristics of the broader PCOS community. Also, priorities for research may be set by researchers, not by people living with the condition. In contrast, the current study is designed to reflect end-user sentiment and does not have any rigid inclusion criteria. Those who post on Reddit are likely to encompass a different, broader population compared to participants included in formal clinical trials (as previous work has demonstrated [17, 18]).

Online data is nevertheless subject to its own unique biases which relate to how those who post choose to use technology [26]. PCOS is associated with a lack of optimised diagnosis and heterogeneity of phenotypes [76]. The most pragmatic general approach to future PCOS research may be to undertake studies using a range of methodologies in different subpopulations, including different geographical and ethnic settings, recognising that all such studies are context dependent and have their own inherent biases and limitations.

The features explored in the final dataset were dependent on the frequency of the features in the manually read data subset and the variable ability of the machine learning to accurately identify specific features. In particular, while there are few ways to mention ‘inositol’ and the machine learning approach is likely to capture this terms very consistently, there are many ways to note ‘exercise’, and thus a higher false negative rate for exercise was expected (Appendix A). While the most frequently discussed features were easily included, some of the less frequent features could not be included without compromising confidence in the conclusions. In Fig. 2, it is apparent that certain features, such as improving fatigue, may have been hidden due to its less frequent discussion within the subreddit. Some features, such as fertility, were identified too infrequently to be included in this study at all. Finally, while the proximity of features mentioned was a good indicator that the features were related, there was always a risk that this analysis could associate two separate ideas together because they are in the same sentence. Attempts were made to mitigate this risk by ignoring conflicting sentiments.

Often users would trial treatments in combination. Therefore, it was possible for the improvement or worsening of their symptoms to be misattributed. The nature of this research made such a risk unavoidable. However, Fig. 3 offered some suggestion as to which treatments unintentionally shared sentiment. Furthermore, when large portions of users were reporting the same sentiment for the same combination, it was unlikely to be due to misattributed sentiment. The analysis of the subreddit cohort is approaching correlation analysis, so attributing causation will always be difficult. However, strictly limiting the proximity of the symptoms and treatments paired to within two sentences provided more confidence in the relationship. Thus, potential for causation should be considered.

In exploratory analyses such as these, there is an increased risk that some of the statistically significant results occurred due to the very wide range of effects considered. Since this research lacked specific hypotheses and acts to test a new form of exploratory medical research, the lack of correction for multiple comparisons could be considered inevitable [77]. Findings from this study should not be considered in isolation or out of context, but rather used to validate existing research or generate hypotheses for future research. For example, this study highlighted the positive sentiment of certain interventions, such as spearmint tea, where the academic literature is very sparse. Furthermore, this study observed frequent positive sentiment regarding a number of interventions emerging in the literature, such as inositol supplementation.

### Usefulness, potential and novelty

Extracting data from a PCOS internet forum to explore treatment perceptions and outcomes has yielded an abundance of interesting findings. Data from the PCOS subreddit captures groups of people who may be under-researched in a clinical setting [17]. These groups include those with potential financial, mobility, and geographic barriers to healthcare access, those who are cynical of the healthcare system, and PCOS phenotypes that do not fit study inclusion criteria. This study has also demonstrated that it is possible to obtain useful and rich clinical data from internet forums using machine learning, as the subreddit data may help inform discussion about treatment options for PCOS. There are hundreds of other internet forums discussing dozens of other medical conditions where such research may be beneficial. Once previously unconfirmed treatment responses can be validated using clinical studies, this research has the potential to expand the treatment options for people with PCOS.

To the authors’ knowledge, no one else has used machine learning to explore internet forums for clinical research and extracted treatment-symptom sentiments as we have. The source of the dataset and means of obtaining it was unique. Furthermore, the authors are unaware of any other clinical dataset that so thoroughly details the perception surrounding different PCOS interventions. This kind of sentiment-driven research can be useful for exploring reported drug side effects in a way that moves beyond classical reporting frameworks. It allows for findings to be drawn from organic real-world data outside of traditional and clinical settings that is difficult to capture otherwise. Furthermore, putting the health condition at the centre of the internet research allows for exploratory descriptive comparisons of differences in side effect profiles between different treatment modalities. As such, the approach allowed for the comparison of a range of different types of interventions.

## Conclusion

Overall, this study has demonstrated that it is possible to derive clinical information from an internet forum. By exploring the PCOS subreddit, a significant amount of useful data was gathered on PCOS intervention strategies and their outcomes. Dieting (RR 5.19, 95% CI 3.28–8.19, n = 99) and intermittent fasting (RR 33.50, 95% CI 8.54–131.34, n = 69) were reported as the most successful interventions for PCOS weight loss, which was supported in the literature [14, 30–33]. Combined oral contraceptive pills were strongly associated with adverse effects such as worsening depression (RR 0.06, 95% CI 0.02–0.25, n = 33), anxiety (RR 0.10, 95% CI 0.03–0.36, n = 23), fatigue (RR 0, n = 45) and low libido (RR 0.03, 95% CI 0.01–0.24, n = 30) within the PCOS subreddit. This result is more controversial, with meta-analyses finding no relationship between contraceptives and mental health [44, 45], while these mental health issues are still commonly reported symptoms [38, 40, 42, 46]. However, combined contraceptives with anti-androgenic progestins had more favourable outcomes. Inositol was associated with a large range of favourable outcomes and was one of the few treatments associated with improved mental health [depression (RR 4.25, 95% CI 1.72–10.51, n = 21), anxiety (RR 5.83, 95% CI 2.76–12.35, n = 41) and mood issues (RR 25.00, 95% CI 3.65–171.10, n = 26)]. Inositol supplementations improving PCOS symptoms is supported within the literature [50, 51], although one review has called into question the quality of key inositol research [52]. The current study highlights the areas of PCOS intervention that could be further explored with clinical research and demonstrates the potential of clinical internet research to explore the pleiotropic effects of a wide range of treatments.

## Appendix

Appendix A – Machine Learning Performance

As reported in previous work, some features utilised machine learning classifiers and the less frequent features relied on search terms [19]. Table

**Table 3:** The different features with network inclusive classification systems

Feature label	Description	Number manually labelled	Number classifier labelled	Precision
Acne	Mention of too much acne on the face or body	661	8525	99
Alopecia	Mention of thinning or balding head hair	574	8366	94
Amenorrhea	Mention of a lack of menstrual cycle for at least three months	587	12,284	84
Anovulation	Mention of a lack of ovulation for at least three months	53	977	71
Anxiety	Mention of struggling with anxiety or experiencing panic attacks	264	5421	99
Bloating	Mention of frequent or severe bloating	116	513	98
COCP	Mention of taking a combined oral contraceptive pill	217	2282	92
Combined contraception pill 1	Mention of taking a specific combined oral contraceptive pill composed of 3 mg of drospirenone and 0.02 mg of ethinyl estradiol	61	1168	92
Cramping	Mention of frequent or severe abdominal cramping	156	4186	98
Cravings	Mention of frequent or severe hunger or cravings	105	1950	90
Dairy free	Mention of trying a dairy free diet	67	1592	92
Depression	Mention of struggling with depression	314	6446	94
Fatigue	Mention of frequent or severe exhaustion or a consistent lack of energy	232	3023	94
Gluten free	Mention of trying a gluten free diet	66	1463	84
Head pain	Mention of frequent or persistent headaches or migraines	111	2300	100
Hirsutism	Mention of too much body or facial hair	975	13,048	95
Inositol	Mention of taking inositol supplements	205	3344	94
Any IUD	Mention of trying a contraceptive intrauterine device	99	2202	96
Ketogenic	Mention of trying a ketogenic diet	199	1454	92
Low calorie	Mention of trying a restricted calorie diet	112	2329	80
Low Carbohydrate	Mention of trying a low carbohydrate diet	253	4149	74
Low libido	Mention of struggling with a low libido	43	707	88
Low sugar	Mention of trying a low sugar diet	99	1879	92
Medroxyprogesterone acetate	Mention of taking medroxyprogesterone acetate	66	1207	94
Menorrhagia	Mention of menstrual bleeding that lasts more than seven days	242	5263	74
Menstrual Irregularities	Mention of experiencing menstrual irregularities	1420	16,168	96
Metformin	Mention of taking metformin	596	7364	97
Mood Issues	Mention of experiencing mood swings or excessive irritability	133	1269	100
Not Overweight	Mention of body weight being in the normal or underweight ranges	308	2267	80
Oligo-Ovulation	Mention of experiencing eight or fewer periods a year or menstrual cycles longer than 35 days	887	11,738	87
Overweight	Mention of body weight being above normal ranges	928	10,161	86
Plant based	Mention of trying a plant based diet	42	1013	92
Polymenorrhea	Mention of menstrual cycles shorter than 21 days	86	2314	80
POP	Mention of trying progesterone only pills	127	2464	96
Spearmint	Mention of trying spearmint supplementation or spearmint tea	49	1166	98
Spironolactone	Mention of taking spironolactone	284	2367	97
Unspecified diet	Mention of trying an unspecified diet	204	4514	90
Unspecified exercise	Mention of trying unspecified exercise	340	6286	76
Vitamin D	Mention of taking vitamin D supplements	50	1206	92
Weight gain	Mention of gaining body weight	555	9695	91
Weight loss	Mention of losing body weight	457	5714	84

^3^ shows the different features with machine learning classification systems and the precision of the corresponding classifier. Table

**Table 4:** The different features with search-based classification systems and the mean accuracy of the search terms

Feature Label	Description	Number Manually Labelled	Number Classifier Labelled	Mean Accuracy of Search Terms
Berberine	Mention of taking berberine supplements	25	646	100
Biotin	Mention of taking biotin supplements	19	456	100
Combined Contraception Pill 2	Mention of taking a specific combined oral contraceptive pill composed of 3 mg of drospirenone and 0.03 mg of ethinyl estradiol	36	386	100
Combined Contraception Pill 3	Mention of taking a specific combined oral contraceptive pill composed of 2 mg of cyproterone acetate and 0.035 of mg ethinyl estradiol	41	405	100
Contraceptive implant	Mention of trying a contraceptive implant	36	797	100
Contraceptive injection	Mention of trying a contraceptive injection composed of medroxyprogesterone acetate	18	415	100
Inflammation	Mention of frequent or severe inflammation	64	1218	99
Intermittent fasting	Mention of trying intermittent fasting or time restricted eating	63	1605	99
Iron	Mention of taking iron supplements	26	744	100
Irritated bowel	Mention of symptoms of an irritated bowel such as bloating, cramping, diarrhoea or constipation	27	771	99
Isotretinoin	Mention of taking isotretinoin	26	172	100
Levonorgestrel IUD	Mention of trying an intrauterine device that releases levonorgestrel	40	935	100
Magnesium	Mention of taking magnesium supplements	21	1013	99
Minoxidil	Mention of taking minoxidil	22	483	100
Omega 3	Mention of taking omega 3 supplements	26	678	100
Probiotic	Mention of taking probiotic supplements	24	470	100
Saw palmetto	Mention of taking saw palmetto supplements	11	291	100
Vaginal ring	Mention of trying a contraceptive vaginal ring	25	291	100

^4^ shows the different features classified using search terms and the corresponding mean accuracy of the search terms on the training dataset. Tables

**Table 5:** The performance of the sentiment network for any features

Label	Sentiment	Testing data			All data		
		Acc	Sen	Spec	Acc	Sen	Spec
Any Feature	Improved	92.2	58.4	97.5	92.6	57.8	98.1
	Worsened	92.3	35.6	99.1	92.2	35.0	99.0

^5^,

**Table 6:** The performance of the sentiment networks for hyperandrogenic features

Label	Sentiment	Testing data			All data		
		Acc	Sen	Spec	Acc	Sen	Spec
Hirsutism	Improved	97.5	62.5	100.0	93.4	35.9	99.7
	Worsened	94.9	25.0	100.0	95.8	26.5	100.0
	Neutral	81.4	91.8	64.4	83.7	91.1	74.2
Acne	Improved	92.3	64.3	100.0	94.3	77.4	99.6
	Worsened	86.2	47.1	100.0	89.7	52.1	98.1
	Neutral	83.1	77.3	86.0	91.9	92.1	91.7
Alopecia	Improved	95.8	50.0	100.0	97.1	80.0	98.9
	Worsened	87.5	50.0	100.0	89.7	73.4	92.9

^6^,

**Table 7:** The performance of the sentiment networks for menstrual features

Label	Sentiment	Testing Data			All Data		
		Acc	Sen	Spec	Acc	Sen	Spec
Menstrual Irregularities	Improved	88.0	54.3	98.6	92.7	61.0	99.2
	Worsened	93.8	14.3	100.0	91.7	11.2	99.5
	Neutral	79.7	55.7	90.8	84.5	74.6	90.1
Oligo-Ovulation	Improved	84.6	38.5	100.0	93.6	68.7	98.8
	Worsened	97.1	25.0	100.0	95.2	14.0	99.9
	Neutral	81.7	64.9	91.0	85.6	75.1	91.3
Amenorrhea	Improved	90.8	33.3	100.0	87.4	10.0	100.0
	Neutral	93.8	60.0	100.0	87.0	67.9	94.6
Menorrhagia	Worsened	91.7	50.0	100.0	89.7	39.0	100.0
	Neutral	79.2	44.4	100.0	90.5	77.8	96.9

^7^,

**Table 8:** The performance of the sentiment networks for metabolic features

Label	Sentiment	Testing Data			All Data		
		Acc	Sen	Spec	Acc	Sen	Spec
Body Weight	Improved	93.3	66.7	100.0	93.2	72.7	98.7
	Worsened	95.5	87.5	97.3	97.1	88.3	99.0
	Neutral	86.6	53.7	96.4	91.3	75.7	96.3
Cravings	Improved	100.0	100.0	100.0	91.8	86.2	93.8
	Worsened	90.9	50.0	100.0	95.5	75.0	98.9
	Neutral	90.9	75.0	100.0	90.9	87.8	92.8

^8^,

**Table 9:** The performance of the sentiment networks for mental health features

Label	Sentiment	Testing Data			All Data		
		Acc	Sen	Spec	Acc	Sen	Spec
Depression	Worsened	86.7	42.9	100.0	88.4	50.8	99.1
	Neutral	93.3	91.7	94.4	92.1	91.9	92.3
Anxiety	Worsened	87.5	25.0	100.0	91.8	63.0	100.0
	Neutral	83.3	76.5	100.0	92.6	91.7	93.4
Mood Irregularities	Improved	100.0	100.0	100.0	97.3	86.7	99.0
	Worsened	81.8	33.3	100.0	90.3	63.3	100.0
	Neutral	90.9	50.0	100.0	92.0	69.0	100.0

^9^,

**Table 10:** The performance of the sentiment networks for other condition based features

Label	Sentiment	Testing Data			All Data		
		Acc	Sen	Spec	Acc	Sen	Spec
Fatigue	Improved	95.8	50.0	100.0	94.8	52.0	100.0
	Worsened	91.7	66.7	100.0	87.9	72.7	94.0
	Neutral	83.3	42.9	100.0	85.8	70.1	95.2
Cramping	Worsened	95.2	100.0	94.1	91.0	75.0	95.2
	Neutral	76.2	75.0	76.9	88.6	73.9	95.7
Bloating	Improved	100.0	100.0	100.0	92.4	66.7	100.0
	Worsened	80.0	33.3	100.0	90.2	62.5	100.0
	Neutral	90.0	66.7	100.0	76.1	26.7	100.0
Bowel Irritation	Improved	93.8	66.7	100.0	96.8	81.5	100.0
	Worsened	93.8	83.3	100.0	93.6	89.5	96.0
	Neutral	81.3	40.0	100.0	87.8	64.7	99.0
Head Pain	Worsened	100.0	100.0	100.0	94.4	89.7	96.5
	Neutral	100.0	100.0	100.0	90.3	85.4	92.8
Low Libido	Improved	93.3	50.0	100.0	98.0	82.4	100.0
	Worsened	86.7	50.0	100.0	92.1	75.0	97.4
	Neutral	93.3	66.7	100.0	94.0	73.1	98.4
Inflammation	Improved	84.6	50.0	100.0	94.9	92.1	96.0
	Worsened	92.3	75.0	100.0	96.4	94.6	97.0
	Neutral	92.3	66.7	100.0	89.9	86.8	91.0

^10^,

**Table 11:** The performance of the sentiment networks for contraceptive features

Label	Sentiment	Testing Data			All Data		
		Acc	Sen	Spec	Acc	Sen	Spec
COCP	Improved	89.2	20.0	100.0	93.4	51.0	100.0
	Worsened	97.3	50.0	100.0	97.8	88.0	98.5
	Neutral	94.6	71.4	100.0	90.2	49.3	99.7
IUD	Improved	95.0	50.0	100.0	97.6	80.0	100.0
	Neutral	90.0	90.0	90.0	91.3	92.5	90.2
Implant	Improved	100.0	100.0	100.0	90.6	55.6	97.7
	Neutral	80.0	50.0	100.0	62.3	4.8	100.0
Combined Contraception Pill 1	Worsened	93.3	75.0	100.0	85.0	32.3	99.1
	Neutral	93.3	75.0	100.0	85.0	77.5	87.9
Vaginal Ring	Improved	84.6	33.3	100.0	93.7	75.0	100.0
	Worsened	100.0	100.0	100.0	96.0	73.7	100.0
	Neutral	76.9	60.0	87.5	90.5	93.9	88.3
Levonorgestrel IUD	Improved	100.0	100.0	100.0	95.2	91.7	96.0
	Worsened	91.7	66.7	100.0	92.0	75.0	96.9
Unspecified Contraception	Improved	100.0	100.0	100.0	75.7	68.4	83.3
	Neutral	100.0	100.0	100.0	86.5	88.2	85.0
Combined Contraception Pill 2	Improved	81.8	33.3	100.0	88.1	62.2	100.0
	Worsened	90.9	66.7	100.0	94.9	82.8	98.9
	Neutral	81.8	75.0	85.7	89.8	77.5	96.2
Combined Contraception Pill 3	Improved	91.7	66.7	100.0	92.4	71.0	100.0
	Worsened	91.7	50.0	100.0	95.0	70.0	100.0
	Neutral	100.0	100.0	100.0	94.1	87.5	98.6
Depo-Provera Injections	Worsened	100.0	100.0	100.0	93.9	84.6	98.7
	Neutral	91.7	75.0	100.0	91.3	79.5	97.4

^11^,

**Table 12:** The performance of the sentiment networks for lifestyle change features

Label	Sentiment	Testing Data			All Data		
		Acc	Sen	Spec	Acc	Sen	Spec
Unspecified Exercise	Improved	93.9	60.0	100.0	93.4	79.2	95.8
	Neutral	78.8	61.5	90.0	91.6	86.0	95.5
Low Carbohydrate	Neutral	91.7	80.0	100.0	94.9	89.0	98.6
Ketogenic	Improved	90.0	50.0	100.0	95.2	79.1	99.4
	Worsened	90.0	33.3	100.0	93.8	62.1	98.9
	Neutral	80.0	42.9	100.0	84.6	54.9	100.0
Low Calorie	Improved	89.5	33.3	100.0	96.4	74.1	100.0
	Neutral	94.7	87.5	100.0	85.0	68.8	95.7
Low Sugar	Improved	94.1	66.7	100.0	97.1	87.1	99.3
	Neutral	76.5	71.4	80.0	90.8	94.0	87.8
Gluten Free	Improved	87.5	33.3	100.0	94.8	75.9	99.2
	Neutral	93.8	85.7	100.0	90.3	87.9	92.1
Dairy Free	Neutral	81.3	50.0	100.0	89.5	77.8	97.8
Plant Based	Neutral	85.7	60.0	100.0	88.9	70.6	98.9
Intermittent Fasting	Improved	100.0	100.0	100.0	87.7	86.8	88.0
	Neutral	92.3	75.0	100.0	79.0	54.2	97.5

^12^,

**Table 13:** The performance of the sentiment networks for prescribed treatment features

Label	Sentiment	Testing Data			All Data		
		Acc	Sen	Spec	Acc	Sen	Spec
Metformin	Improved	91.2	50.0	98.3	91.8	47.5	99.3
	Worsened	95.6	40.0	100.0	94.1	45.3	99.2
	Neutral	82.4	82.9	81.8	89.1	87.6	90.5
Spironolactone	Improved	91.2	25.0	100.0	84.5	12.1	99.6
	Worsened	91.2	25.0	100.0	95.2	71.4	98.0
	Neutral	91.2	66.7	96.4	83.0	51.0	97.4
Isotretinoin	Improved	75.0	25.0	100.0	90.8	67.6	100.0
	Worsened	91.7	50.0	100.0	90.8	54.5	99.0
	Neutral	91.7	80.0	100.0	88.2	73.5	98.6
Phentermine	Improved	91.7	100.0	88.9	93.8	100.0	91.9
	Worsened	100.0	100.0	100.0	92.9	83.3	94.7
	Neutral	91.7	100.0	85.7	90.2	91.4	88.9

^13^,

**Table 14:** The performance of the sentiment networks for supplement features

Label	Sentiment	Testing Data			All Data		
		Acc	Sen	Spec	Acc	Sen	Spec
Inositol	Improved	96.2	100.0	94.7	95.5	85.3	99.0
	Worsened	96.2	50.0	100.0	97.4	53.3	100.0
	Neutral	88.5	75.0	100.0	95.5	93.2	97.3
Spearmint	Worsened	100.0	100.0	100.0	98.4	70.0	100.0
	Neutral	80.0	75.0	83.3	93.2	91.5	94.5
Vitamin D	Neutral	94.7	100.0	87.5	90.1	94.8	82.7
Magnesium	Improved	100.0	100.0	100.0	96.7	83.3	99.0
	Neutral	91.7	100.0	66.7	74.4	90.7	34.3
Probiotic	Improved	100.0	100.0	100.0	89.8	55.6	100.0
	Neutral	90.9	100.0	75.0	95.8	96.3	94.6
Omega 3	Improved	93.8	66.7	100.0	95.8	80.6	99.3
	Neutral	62.5	45.5	100.0	53.0	49.2	62.5
Iron	Improved	91.7	50.0	100.0	94.8	71.4	100.0
	Neutral	100.0	100.0	100.0	91.3	94.7	85.0
Biotin	Improved	91.7	50.0	100.0	95.7	79.2	100.0
	Neutral	83.3	100.0	50.0	90.4	100.0	70.3
Saw Palmetto	Improved	100.0	100.0	100.0	94.6	89.7	96.3
	Neutral	90.9	100.0	80.0	92.8	95.2	89.6
Berberine	Improved	100.0	100.0	100.0	90.8	72.7	95.4
	Neutral	63.6	42.9	100.0	58.7	48.6	76.9

^14^ show the performance of the networks used to classify sentiment.
